# APS8 Delays Tumor Growth in Mice by Inducing Apoptosis of Lung Adenocarcinoma Cells Expressing High Number of α7 Nicotinic Receptors

**DOI:** 10.3390/md16100367

**Published:** 2018-10-03

**Authors:** Sabina Berne, Maja Čemažar, Robert Frangež, Polona Juntes, Simona Kranjc, Marjana Grandič, Monika Savarin, Tom Turk

**Affiliations:** 1 Department of Biology, Biotechnical Faculty, University of Ljubljana, Večna pot 111, SI-1000 Ljubljana, Slovenia; sabina.berne@bf.uni-lj.si; 2 Department of Experimental Oncology, Institute of Oncology Ljubljana, Zaloška 2, SI-1000 Ljubljana, Slovenia; mcemazar@onko-i.si (M.Č.); skranjc@onko-i.si (S.K.); msavarin@onko-i.si (M.S.); 3 Faculty for Health Sciences, University of Primorska, Polje 42, SI-6310 Izola, Slovenia; 4 Institute of of Preclinical Sciences, Veterinary Faculty, University of Ljubljana, Gerbičeva 60, SI-1000 Ljubljana, Slovenia; robert.frangez@vf.uni-lj.si; 5 Institute of Pathology, Wild Animals, Fish and Bees, Veterinary Faculty, University of Ljubljana, Gerbičeva 60, SI-1000 Ljubljana, Slovenia; polona.juntes@vf.uni-lj.si; 6 Institute of Food Safety, Feed and Environment, Veterinary Faculty, University of Ljubljana, Gerbičeva 60, SI-1000 Ljubljana, Slovenia; marjana.grandic@vf.uni-lj.si

**Keywords:** lung cancer, antitumor activity, A549, HT29, CHRNA7, alkylpiridinium, SCID mice, toxicity, apoptosis

## Abstract

The alkylpyridinium polymer APS8, a potent antagonist of α7 nicotinic acetylcholine receptors (nAChRs), selectively induces apoptosis in non-small cell lung cancer cells but not in normal lung fibroblasts. To explore the potential therapeutic value of APS8 for at least certain types of lung cancer, we determined its systemic and organ-specific toxicity in mice, evaluated its antitumor activity against adenocarcinoma xenograft models, and examined the *in-vitro* mechanisms of APS8 in terms of apoptosis, cytotoxicity, and viability. We also measured Ca^2+^ influx into cells, and evaluated the effects of APS8 on Ca^2+^ uptake while siRNA silencing of the gene for α7 nAChRs, *CHRNA7*. APS8 was not toxic to mice up to 5 mg/kg i.v., and no significant histological changes were observed in mice that survived APS8 treatment. Repetitive intratumoral injections of APS8 (4 mg/kg) significantly delayed growth of A549 cell tumors, and generally prevented regrowth of tumors, but were less effective in reducing growth of HT29 cell tumors. APS8 impaired the viability of A549 cells in a dose-dependent manner and induced apoptosis at micro molar concentrations. Nano molar APS8 caused minor cytotoxic effects, while cell lysis occurred at APS8 >3 µM. Furthermore, Ca^2+^ uptake was significantly reduced in APS8-treated A549 cells. Observed differences in response to APS8 can be attributed to the number of α7 nAChRs expressed in these cells, with those with more AChRs (i.e., A549 cells) being more sensitive to nAChR antagonists like APS8. We conclude that α7 nAChR antagonists like APS8 have potential to be used as therapeutics for tumors expressing large numbers of α7 nAChRs.

## 1. Introduction

Many cancer and nonneuronal normal cells express different subtypes, numbers and combinations of nicotinic acetylcholine receptors (nAChRs) [1,2,3]. These pentameric ligand-gated ion channels selective for cations [4] are activated by acetylcholine (ACh) and regulate diverse cellular processes such as proliferation, differentiation, cell-cell interaction, and apoptosis in autocrine or paracrine manners [5,6]. Nicotine and its derivatives, 4-(methylnitrosamino)-1-(3-pyridyl)-1-butanone (NNK) and N-nitrosonornicotine (NNN), have high affinity for nAChRs and can displace ACh from these receptors, thus disrupting normal cell responses to ACh signaling [3]. Exposure to nicotine and its derivatives changes the expression pattern of nAChRs subtypes in the lung [7], colon [8], breast [9], pancreatic [10], and urinary bladder cancer cell lines [11], inducing cell proliferation, tumor growth and metastasis [12]. Additionally, numerous reports have associated nicotine and its derivatives with a greater risk for the development of small-cell and non-small-cell lung carcinomas [13,14], as well as gastric [6], pancreatic [15], colon [8], breast [9], and urinary bladder cancers [6].

The binding of nicotine and its derivatives to nAChRs activates cell signaling pathways that prevent apoptosis, enhance cell proliferation, stimulate migration and promote angiogenesis [16,17,18,19]. These effects support tumor growth and metastasis. They involve mainly the α7 and α9 nAChRs, with possible contribution from the β-adrenergic receptors and/or epidermal growth factor receptors [12]. In lung cancer, elevated cytosolic Ca^2+^ concentration following the activation of the α7 nAChR initiates multiple downstream cell signaling cascades, including the MAPK/ERK, JAK/STAT and PI3K/AKT signaling pathways [12,17]. Another cascade involves binding of the β-arrestin-1 that recruits Src kinase. Kinase activates Raf-1 cascade [20] and subsequently contributes to the activation of the Rb-E2F pathway [12]. Binding of ligands to the nAChR also activates several transcription factors, including AP1, which is responsible for the overexpression of the α9 nAChR in breast cancer cells. The overexpression of these particular receptors makes them more susceptible to the proliferative effects of nicotine and other agonists [21].

Binding of antagonists to nAChRs should have the opposite effects. Unfortunately, only a few such competitive nAChR antagonists are currently known, and these are either highly toxic or not specific (e.g., α-bungarotoxin, α-cobratoxin, and methyllycaconitine, respectively). Furthermore, the mechanisms that lead to apoptosis upon binding of such nAChR antagonists are not well understood, and there have been some controversies whether nAChR antagonists might have any role in lung cancer treatment [22,23,24].

**Figure 1:**
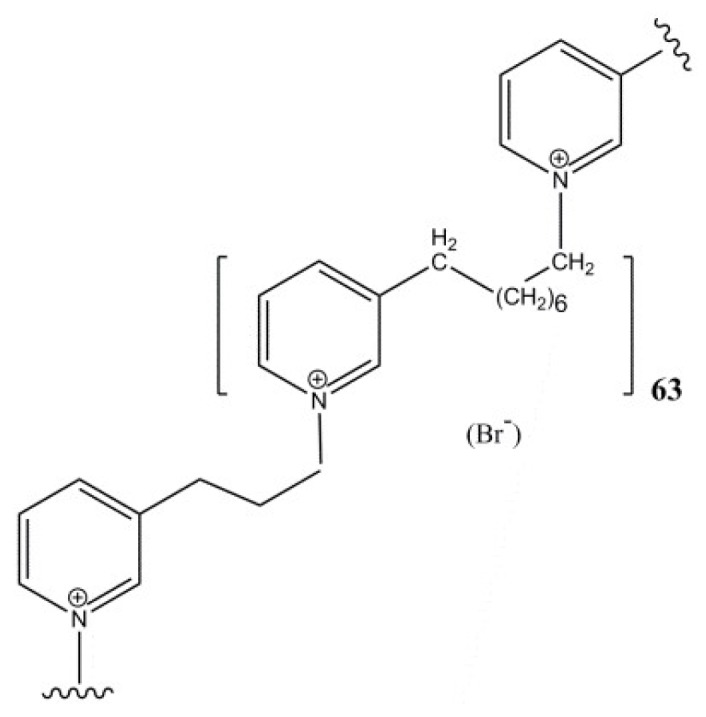
Chemical structure of APS8.

Natural alkylpyridinium polymers (polyAPS), isolated from the marine sponge *Haliclona* [*Rhizoniera*] *sarai* [25] have several biological activities, including acetylcholinesterase inhibition [26,27]. Based on these natural polyAPS, synthetic alkylpyridinium compounds with similar structural and functional properties have been synthesized [28,29]. One of these analogs, APS8 (Figure 1) binds to the α7 nAChR and completely blocks its activity at 1–3 nM APS8, but is less effective in blocking the α4β2 nAChR. We have previously shown that APS8 can considerably attenuate the anti-apoptotic effects of nicotine in a human non–small-cell lung carcinoma cell line [30]. In addition, APS8 selectively kills cancer cells, while normal lung fibroblasts are essentially not affected. Binding of APS8 to the α7 nAChR results in the promotion of the intrinsic apoptotic pathway through the activation of pro-apoptotic factors and the blocking of anti-apoptotic factors [30].

To further explore the potential therapeutic efficacy of APS8, we evaluated the mechanisms of APS8 activity in vitro and its toxicity and antitumor effects in vivo. Specifically, we investigated the in vitro activity of APS8 by monitoring apoptosis, cytotoxicity, and cell viability through Ca^2+^ influx measurements. We also studied the effects on Ca^2+^ uptake by silencing *CHRNA7,* the gene that encodes α7 subunit which forms the α7 homopentameric nAChR. Systemic toxicity and organ-specific toxicity of APS8 were determined in vivo, together with evaluation of the antitumor potential of APS8 through measurements of tumor growth and determination of necrosis, apoptosis, and cell proliferation in tumor sections.

## 2. Results

### 2.1. Mechanism of APS8 Toxicity

**Figure 2:**
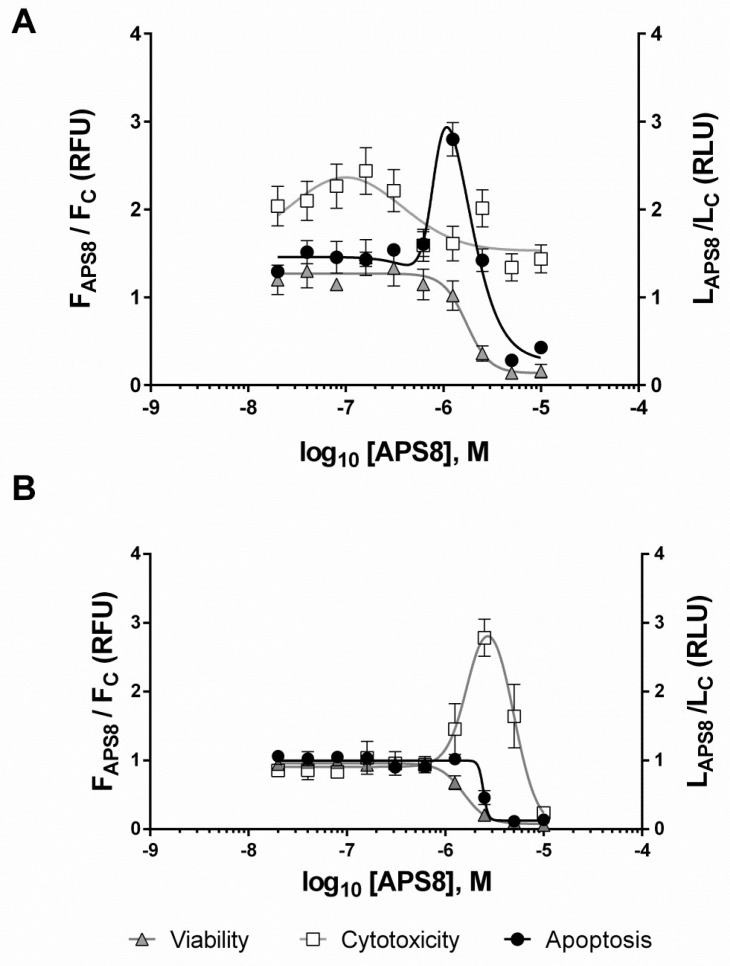
Dose-response curves for APS8-treated A549 cells (**A**) and HT29 cells (**B**). Cell viability and cytotoxicity were measured after 6 h treatment with APS8, while apoptosis was assessed after 8 h. Data are background-corrected means  ± SEM (*n* = 4–8), normalized to the untreated cells. F, fluorescence; L, luminescence.

The effects of APS8 on A549 and HT29 cells were studied in single assay wells in terms of their viability, the APS8 cytotoxicity, and caspase 3/7 activity. APS8 treatment for 6 hours impaired the viability of the A549 cells in a dose-dependent manner with an estimated EC_50_ of 1.7 µM (Figure 2A). The minor cytotoxic effects of nanomolar APS8 were attributed to the formation of transient pores in the cell membranes [31,32], while higher concentrations of APS8 (>3 µM) likely caused primary necrosis by detergent-like mechanisms of cell lysis (Figure 2A). After 8 h of incubation, there was an increase in caspase 3/7 activity (Figure 2A). This effect was consistent with apoptosis and was seen in a narrow range of APS8 concentrations from 0.3 µM to 2.5 µM, with an estimated EC_50_ between 0.8 µM and 1.6 µM APS8. APS8 treatment resulted in a dose-dependent decrease in viability of the HT29 cells (Figure 2B) with an estimated EC_50_ of 1.5 µM. In contrast to A549 cells, APS8 caused an increase in cytotoxicity of HT29 cells with no caspase 3/7 activation (Figure 2B), which is consistent with primary necrosis [33].

### 2.2. Ca^2+^ Imaging

**Figure 3:**
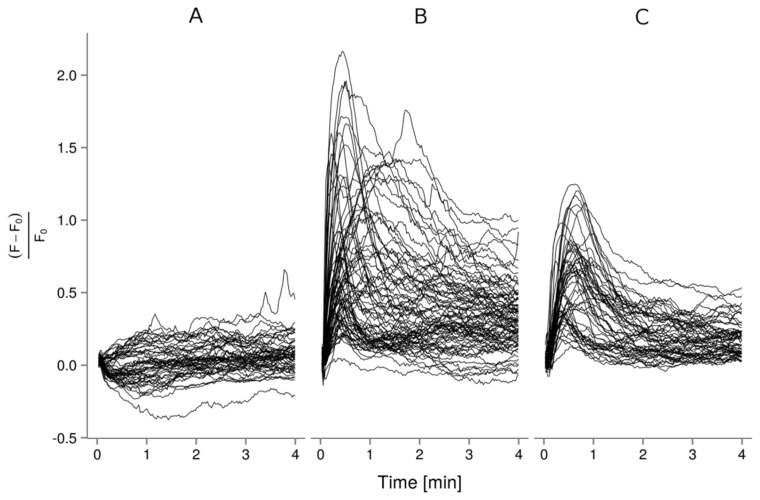
Time courses of Ca^2+^ uptake in individual A549 cells. Cells were treated with 600 nM APS8 (**A**), 6 mM AR-R17779 (**B**), or their combination (**C**). Data are relative fluorescence units as fractional differences in fluorescence intensity of the Ca^2+^ indicator dye Fluo-4 post (F) and prior (F_0_) to APS8 application.

Using confocal microscopy and the fluorescent Ca^2+^ indicator probe Fluo-4, Ca^2+^-uptake was investigated in individual A549 cells initially with APS8 treatment (Figure 3A). Here we observed slight increase of cytosolic Ca^2+^ level, indicating the possible formation of transient pores in the cell membrane [31,32]. The extensive Ca^2+^ influx was evident after activation of the α7 nAChRs by the highly selective agonist AR-R17779, which is a conformationally restricted analog of ACh (Figure 3B). However, when A549 cells were preincubated with APS8 and then challenged by AR-R17779 (Figure 3C), the fluorescence signal due to the Ca^2+^ uptake was significantly lower (*p* = 0.019, Mann–Whitney U test). 

**Figure 4:**
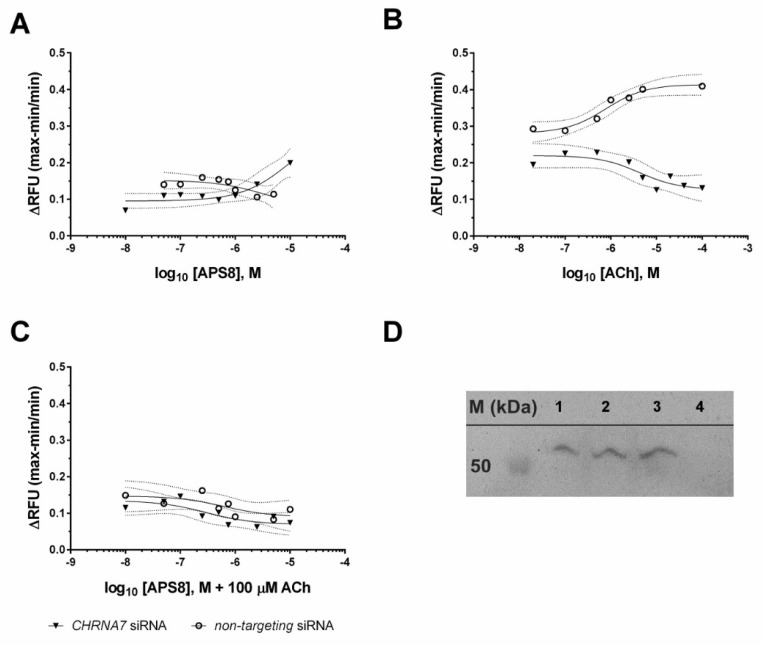
Dose-response curves of Ca^2+^ uptake in A549 cells and Western blot panel showing CHRNA7 gene silencing. Cells were treated with various concentrations of APS8 (**A**; 10, 5, 2.5, 1, 0.75, 0.5, 0.25, 0.1, 0.05, 0.01 µM) or ACh (**B**; 100, 40, 20, 10, 5, 2.5, 1, 0.5, 0.1, 0.02 µM) or preincubated (20 min, RT) with various concentrations of APS8 and then treated with 100 µM ACh (**C**). The A549 cells were silenced with CHRNA7-targeted or nontargeting (negative control) siRNAs. Data are relative fluorescence units (RFU), as maximum response minus minimum response, divided by the minimum response. The curves and their 95% confidence intervals were obtained by fitting the data with nonlinear regression function (GraphPad Prism). Recombinant human α7 nAChR (#H00001139-P01, Abnova; Lane 1) and lysates of A549 cells treated with nontargeting siRNA (Lane 2) or siRNA targeting CHRNA7 (Lanes 3 and 4) were separated by SDS-PAGE followed by Western blotting (**D**) using rabbit antibodies against human α7 nAChR (1:500; #HPA029422, Sigma-Aldrich) and Peroxidase AffiniPure Goat Anti-Rabbit IgG (1:10,000; #111-035-003, Jackson ImmunoResearch Europe Ltd).

Ca^2+^ uptake was then measured in a microplate reader using the fluorescent Ca^2+^ indicator dye Fluo-4 and physiological agonist of nAChR, acetylcholine. Here we also determined the responses of A549 cells which have been CHRNA7 silenced using siRNA. Such cells have down-regulated expression of α7 nAChR, as compared to the cells transfected by nontargeting siRNA. When APS8 was applied, there were no significant changes in Ca^2+^ influx up to 3 µM APS8 (Figure 4A), at which concentration APS8 presumably disrupts cell membrane integrity and induces transient pores that allowed calcium influx. The silencing of the α7 nAChR expression considerably decreased Ca^2+^ uptake by ACh, with an estimated EC_50_ of 4.2 µM ACh as compared to the estimated EC_50_ of 0.78 µM ACh for the non-targeting negative control siRNA (Figure 4B). Furthermore, after exposing the cells to various concentrations of APS8 for 20 min and then stimulating them with 100 µM ACh (Figure 4C) there were no significant changes in the fluorescence signal. We concluded that APS8 acts as an α7 nAChR antagonist. 

### 2.3. Determination of LD_50_ in Mice

The APS8 toxicity in mice was examined, with the determination of the LD_50_ in a mouse lethality assay. The i.v. LD_50_ of APS8 in Balb/c mice was 6 mg/kg. There were no signs of mice intoxication at the dose of 3 mg/kg APS8, while at 5 mg/kg and 6 mg/kg APS8, two mice survived and completely recovered shortly afterwards (5–10 min), but one died within 3 min. At the dose of 7 mg/kg APS8 and higher, there were signs of intoxication (cyanosis, cessation of movement, hair bristling) and all of the animals tested at this dose died in <5 min. The surviving mice from all of the treatment groups were sacrificed 24 h after the APS8 treatment. 

The histological examination of these mice revealed no lesions related to the application of APS8 in the myocardium, spleen, and lung. There was mild centrolobular macrovesicular fatty infiltration in the liver, but with no clear difference among the groups or between the mice that died immediately and those that were later sacrificed. We noted rare disseminated necrotic hepatocytes in the liver of two out of the three mice from the group that received 3 mg/kg APS8, but not in the other mice that had survived. In one mouse that survived for 24 h after receiving an APS8 dose of 5 mg/kg, and in one mouse which received 6 mg/kg APS8, there was severe diffuse tubular necrosis in the deep cortex of the kidney, with mild acute multifocal inflammatory reactions in necrotic areas visible in the first mouse (Figure S2*.* Panels C and D). There was little necrotic or apoptotic cell shedding into the tubular lamina in the kidneys of the mice treated with 3 mg/kg (3 of 3) and 6 mg/kg (2 of 3) that survived 24 h, as well in 1 of 3 mice that died immediately after receiving 5 mg/kg and 7 mg/kg, respectively, albeit with no specific differences. We found no such lesions in kidneys of the SCID mice treated by a single or six intratumoral doses of APS8 (4 mg/kg). 

### 2.4. Antitumor Effectivness

We observed pronounced antitumor effects in A549 tumors after intratumoral injection of APS8 at 4 mg/kg. The growth of these tumors was delayed for 22.5 ± 2.7 days compared to the control group (tumor doubling time = 6.3 ± 0.4 days). On the other hand, systemic administration and intratumoral treatment with a lower dose of APS8 (2 mg/kg) did not result in significant effects (Figure S3). As a parameter of toxicity, the body weight changes of SCID A549 tumor bearing mice were monitored and no changes in the mouse body weight were observed (Figure S4).

**Figure 5:**
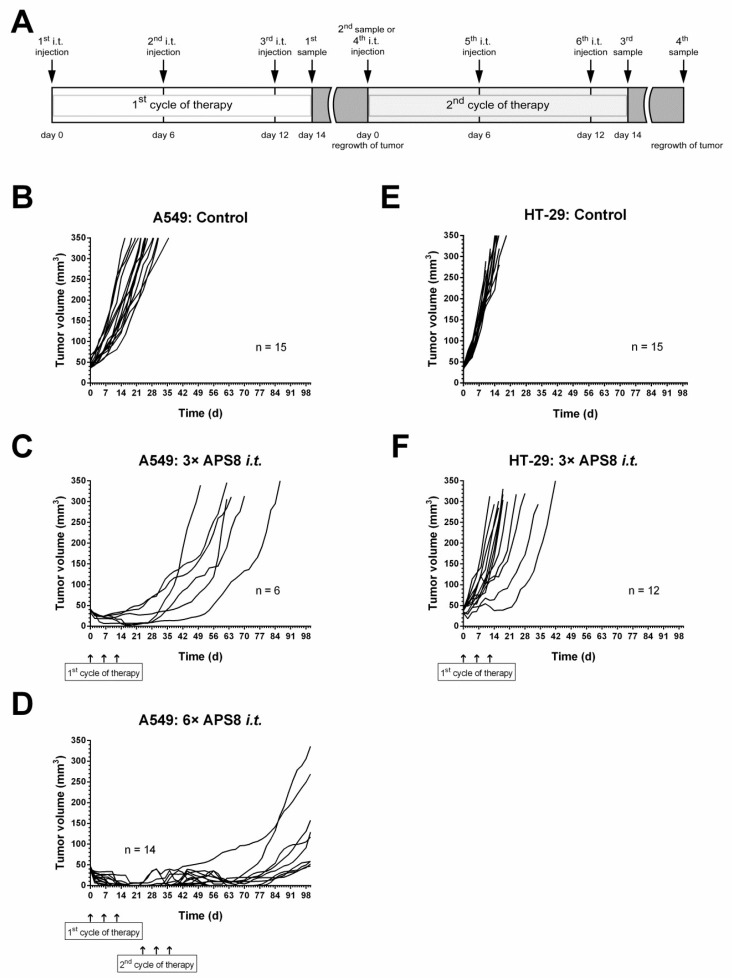
APS8 application regime and tumor growth curves. Treatment protocols for the intratumoral (i.t.) injection of APS8 in the A549 xenograft lung adenocarcinoma and HT29 colon cancer models (**A**). Growth curves after intratumoral injections of APS8 (4 mg/kg) in the A549 xenograft lung adenocarcinoma (**B**–**D**) and HT29 colon adenocarcinoma (**E**,**F**) model, shown individually for each tumor. Tumors were treated as the controls (**B**,**E**) or with either one cycle of APS8 treatment (**C**,**F**; three intratumoral APS8 injection) every 6 days, or two cycles of APS8 treatment (**D**) for the A549 tumors that regrew after the first cycle.

In the second part of the study, repetitive intratumoral injections of 4 mg/kg APS8 were evaluated. After the first cycle of treatment, all of the tumors regressed, although they started to regrow after ~30 days from the start of the treatment. The second treatment cycle of 4 mg/kg APS8 was therefore performed, which stopped the growth of the tumors in 9/14 of the mice, with five (36%) of the tumors showing complete regression, resulting in tumor-free mice for up to 100 days (Figure 5). 

Repetitive intratumoral treatment of HT29 colon adenocarcinoma tumors resulted in minor, but statistically significant growth delays. We postulate that these differences in the tumor responses to APS8 were due to the differences in the expression of α7 nAChR in HT29 and A549 tumors (Figure S5).

**Figure 6:**
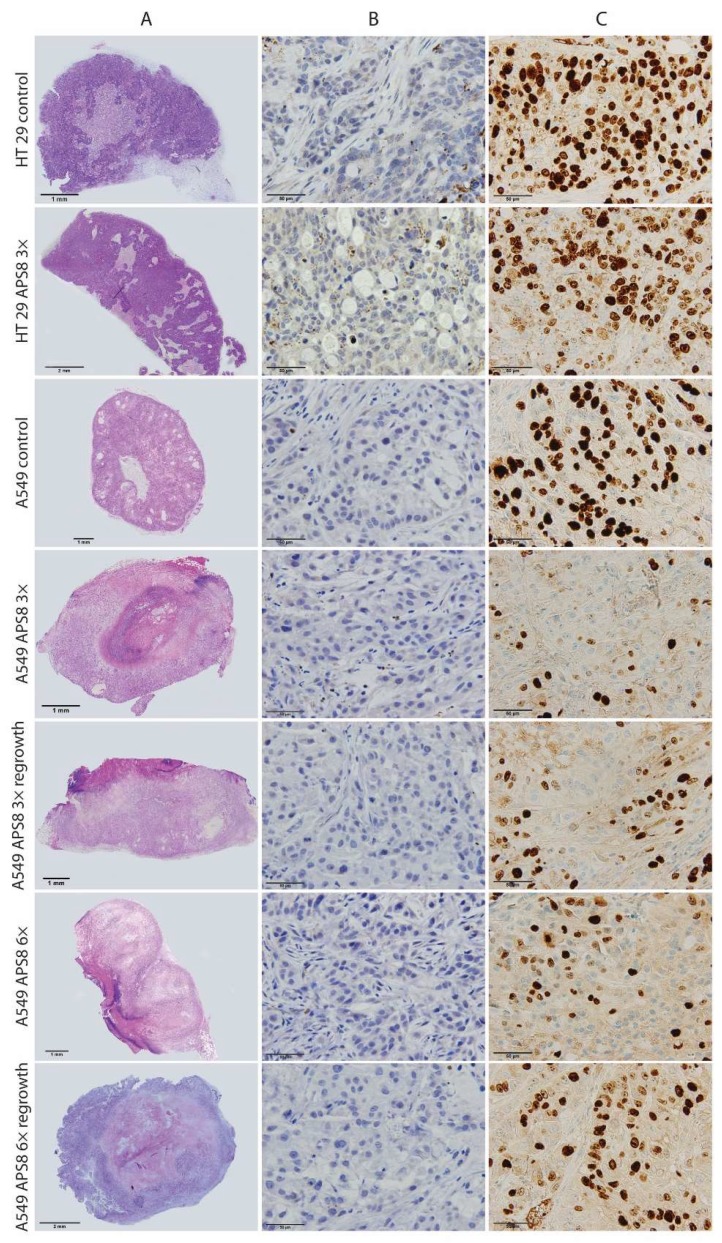
Tumor histology after intratumoral APS8 treatment. Representative images of histology of human A549 xenograft lung adenocarcinoma or HT29 colon adenocarcinoma tumors treated with intratumoral injections of APS8 (as indicated). Row **A**: Tumors stained with hematoxylin and eosin for evaluation of necrosis. Row **B**: Tumors stained for caspase-3 for evaluation of apoptotic cells (brown). Row **C**: Tumors stained for Ki-67 for evaluation of proliferation status. Brown nuclei, proliferative cells.

Based on the histological analysis, the HT29 tumor model was a highly proliferative tumor, with up to 70% Ki-67–positive cells (Figure 6). There was no reduction in proliferating cells after the intratumoral injection of APS8 in the HT29 tumors. Furthermore, the percentage of apoptotic cells and necrotic regions were both in the same range as for the control untreated tumors. 

**Figure 7:**
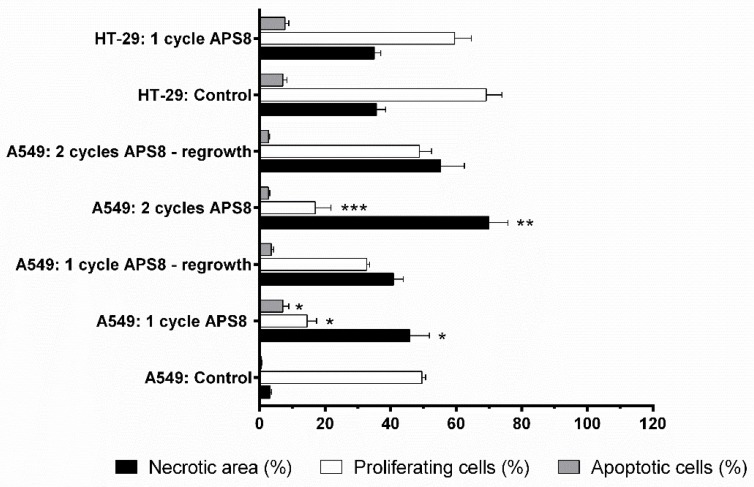
Determination of tumor necrotic area and percentage of apoptotic and proliferating cells after APS8 treatment. Quantification was done from histological sections of human A549 xenograft lung adenocarcinoma and HT29 colon adenocarcinoma tumors after intratumoral treatment with APS8, as one or two cycles of treatment. Data are means ± SEM (*n* = 3). Necrotic areas evaluated on whole tumor sections. Percentages of apoptotic cells or proliferative cells were estimated in five fields in viable tumor areas. * *P* < 0.05, for A549 tumors untreated *versus* after one cycle of APS8 treatment; ** *P* < 0.05, for A549 tumors that responded to APS8 treatment *versus* those that started to regrow; *** *P* < 0.05, for A549 tumors treated with one *versus* two cycles of APS8 treatment.

Compared to the HT29 tumor model, the A549 tumor model was less proliferative. After the first cycle of APS8 therapy, there were fewer proliferating cells in the A549 tumors, compared to the untreated tumors. There was a significantly higher tumor necrosis in the A549 tumors, compared to the control group, and a significant increase in the numbers of apoptotic cells. In tumors that started to regrow after three applications of APS8, the proliferative fraction of cells increased significantly; however, the second cycle of APS8 treatment led to significant reductions in the proliferating cells, which were at similar levels as after the first cycle of APS8 therapy. This indicates that the cells that regrew did not develop resistance. Again, the number of apoptotic cells increased minimally and necrosis was the predominant route of cell death after the APS8 treatments. In the tumors that regrew after the second cycle of APS8 treatment, the fraction of proliferative cells in the viable tumor tissue was similar to the control (untreated tumors), while necrotic regions were still present in these tumors (Figure 6 and Figure 7).

## 3. Discussion

Recently there has been some controversy due to conflicting data considering the use of nAChR antagonists for lung cancer treatment. Most notably, although the studies of Grozio et al. [34] and Paleari et al. [22,35] showed successful treatment with a long-chained α-cobratoxin of human lung adenocarcinoma grafted directly into the NOD/SCID mouse lung, this finding was later refuted by Alama et al. [23]. They repeated the study using the same protocols and reported no obvious effects in terms of tumor growth reduction. In addition, they also used a short chain α-cobrotoxin that even enhanced the tumor growth, albeit without reaching statistical significance [23]. Despite these conflicting data, different neuronal subtypes of the nAChR that are expressed in large numbers in certain cancer cells remain in the focus of many studies, as potential targets for cancer treatments and the development of new anticancer drugs [22,36,37]. 

Nevertheless, the possible efficient treatment of tumors in experimental models using snake α-toxins remains questionable and problematic due to their high toxicity and predominant effects on muscle nAChRs. Therefore, there is the need for less toxic nAChR antagonists with higher specificity and less side effects. Our previous study revealed that the synthetic analog APS8 that is based on natural alkylpyridinium polymers obtained from a marine sponge caused apoptosis and inhibited proliferation of A549 cells and SK-MES squamous carcinoma cells in vitro [30]. APS8 is a noncompetitive antagonist of the α7 nAChR that completely blocks this receptor type at 1 nM to 3 nM, but is substantially less effective in blocking the α4β2 nAChR. Inhibition of α7 nAChR by APS8 caused apoptosis in both cell types due to the induction of intrinsic pro-apoptotic signaling, as reflected by the up-regulation of pro-apoptotic factors such as caspase 3, Bad, Cyt c, and SMAC, and by the down-regulation of anti-apoptotic factors like BCl-2, XIAP, and survivin [30].

A large number of studies have linked the activation of different nAChR subtypes by nicotine and its derivatives, such as NNK and NNN, to the downstream Ca^2+^ signaling that leads to cell survival, proliferation, growth and angiogenesis [2,6,16,17,18,19,20]. nAChR activation results in increased inward flux of several cations. In this regard, the α7 nAChR is of particular importance, due to its high permeability to Ca^2+^. The α7 nAChR is about 4-fold more permeable to Ca^2+^compared to all of the other nAChR subtypes [38]. The elevated intracellular Ca^2+^ concentration depolarize the cell membrane, which triggers the opening of voltage-gated Ca^2+^ channels and results in further increases in intracellular Ca^2+^ levels [39]. Apoptosis is repressed by Ca^2+^-induced activation of the PI3K/Akt signaling pathway. Akt phosphorylates and inactivates pro-apoptotic factors like Bad and Bax, followed by recruitment of anti-apoptotic factors like XIAP/survivin, and nuclear factors like NF-κB, that enhance cell survival and proliferation [17].

Using confocal microscopy and Ca^2+^ imaging, we showed that APS8 significantly reduces the influx of Ca^2+^ that is triggered by AR-R17779, a highly selective agonist of the α7 nAChR. This suggests specific binding of APS8 to the α7 nAChR and reduced Ca^2+^flux into the cell. Hence this indicates that APS8 probably acts as an antagonist of the α7 nAChR, which corroborate the data obtained by Zovko et al. [30].

The experiments on the *CHRNA7*-silenced cells also support the data obtained by confocal microscopy. Ca^2+^ influx was not increased in the *CHRNA7*-silenced cells upon addition of ACh, as compared to the control cells. Furthermore, the Ca^2+^ influx was not restored by ACh in the cells that had been preincubated with APS8. We should mention that at higher APS8 concentrations (>10^−6^ M) there was a small increase in Ca^2+^ influx in the *CHRNA7*-silenced cells, which was probably due to the induction of transient membrane pores. However, this effect was not observed in the nontargeting siRNA negative controls.

The present study also used SCID mice and subcutaneously grafted A549 tumors. These experiments revealed that APS8 inhibits tumor growth following its intratumoral injection at 4 mg/mL without any toxic side effects. When the same dose was injected intravenously, this did not produce any notable antitumor effects, which indicated that the intratumoral concentrations of APS8 reached after the intravenous injections were not high enough. Furthermore, we can confirm a positive correlation between the expression of α7 nAChRs and APS8, as HT29 cells, which express negligible amounts of the α7 nAChR, treated with APS8 for 8 h did not show induction of caspase 3/7 activity and also APS8 was not effective against HT29 tumors, as compared to the A549 cells. In addition, in the second part of the in vivo study, we demonstrated that these tumors did not develop resistance to the APS8 application. The second cycle of the APS8 therapy, which was performed when the tumors started regrowth after the first cycle (i.e., ~30 days after the first cycle), demonstrated equal or even higher efficacy of APS8, with 36% long-term tumor regression. 

Histological analysis of tumor sections revealed highly necrotic areas in the APS8-treated tumors, and only minor increases in the fractions of apoptotic cells. Therefore, a direct cytolytic activity of APS8 when applied intratumorally cannot be entirely excluded. It is known that above certain concentrations, alkylpyridinium polymers can cause direct membrane damage [28,32]. Proliferation of tumor cells was also reduced after both cycles of APS treatment; however, in the tumors that regrew after the second cycle, the fraction of proliferating cells increased again, which indicated the loss of APS8 inhibitory effects on the α7 nAChR signaling pathway in certain percentage of tumors. 

## 4. Materials and Methods 

Ethics approval: The experiments in BALB/c mice were approved by the Veterinary Administration of the Republic of Slovenia (VARS; Permit no. 34401-84/2008/6). The experiments in SCID mice were approved by the VARS (Permit no. 34401-44/2012/3).

### 4.1. Cell Culture

The A549 human lung adenocarcinoma cells and HT29 human colon adenocarcinoma cells were all from the European Collection of Cell Cultures (UK). The cells were cultured according to the supplier recommendations, in a humidified 5% (*v*/*v*) CO_2_ atmosphere at 37 °C.

### 4.2. APS8 Stock Solution

APS8 is a synthetic analog of 3-alkylpyridinium salts with a molecular weight of 11.9 kDa [29]. The stock solution was prepared in deionized water at 0.84 mM, and was stored at +4 °C.

### 4.3. Transient SiRNA Silencing and Quantitative Real-Time PCR Confirmation of Knock-Down

To knock-down the α7 homopentameric nAChR, the expression of the α7 subunit was down-regulated using silencing small-interfering (si)RNAs directed against the CHRNA7 transcript. Thus, two Silencer^®^ Select Pre-Designed siRNAs were used that target human CHRNA7 (i.e., #s3053, #s3054), along with a positive siRNA control that targets human GAPDH (Silencer^®^ Select GAPDH Positive Control siRNA), and a nontargeting negative control siRNA (Silencer^®^ Select Negative Control #2 siRNA) (all from Ambion^®^, Thermo Fisher Scientific, Waltham, MA, USA). The A549 cells were reverse transfected in triplicate with Lipofectamine^®^ RNAiMAX Transfection Reagent (Invitrogen, Thermo Fisher Scientific, Waltham, MA, USA) and the siRNAs. Twenty-four hours after transfection, the cells were harvested for the Ca^2+^ uptake experiments. After each transfection, the siRNA silencing of CHRNA7 was verified using quantitative real-time (qRT)-PCR and Western blotting (Figure S1. Panels A and B). For the qRT-PCR, the cells were lysed and reverse transcribed with Cells-to-C_T_™ kits (Ambion^®^, Thermo Fisher Scientific, Waltham, Ma, USA). The efficiency of the siRNA treatments was then measured as two biological and two technical replicates using a RT-PCR system (7900HT Fast; Applied Biosystems, Thermo Fisher Scientific, Waltham, Ma, USA) with TaqMan chemistry. Gene expression assays (Applied Biosystems, Thermo Fisher Scientific, Waltham, MA, USA) were carried out for the transcripts of interest: CHRNA7 (Hs01063372_m1); the positive siRNA control GAPDH (Hs99999905_m1); and the endogenous controls of ACTB (β-actin; Hs03023880_g1), TOP1 (topoisomerase I; Hs00243257_m1), SF3A1 (splicing factor 3a, subunit 1; Hs01066327_m1) and RPPH1 (ribonuclease P RNA component H1; Hs03297761_s1). Relative quantification was through the software tools of SDSv2.4.1, RQ Manager and DataAssist^TM^v3.01 (Applied Biosystems, Thermo Fisher Scientific,Waltham, MA, USA). We calculated the fold-changes [40] for the CHRNA7 and GAPDH transcript levels (normalized to the expression of the endogenous controls) in the A549 cells treated with the specific siRNA and the nontargeting, negative control, siRNA [41]. The percentage of the remaining transcript was determined according to the Applied Biosystems application notes (Publication 127AP07-02).

### 4.4. Protein Extraction and Western Blotting 

Total cellular proteins were extracted in Roche cOmplete™ Lysis-M buffer with protease inhibitors, and also supplemented with Roche PhosSTOP™ phosphatase inhibitors (both from Sigma-Aldrich, St. Louis, MO, USA). Whole cell lysates were sonicated on ice and the insoluble material was removed by centrifugation. We determined the protein concentrations using Pierce™ BCA Protein Assay kits (Thermo Fisher Scientific). Equal amounts of protein were separated by SDS-PAGE, along with a recombinant human α7 nAChR (#H00001139-P01, Abnova, Jhongli, Taiwan) as a positive control. This was followed by Western blotting with rabbit antibodies against human α7 nAChR (1:500; #HPA029422, Sigma-Aldrich) and Peroxidase AffiniPure Goat Anti-Rabbit IgG (1:10,000; #111-035-003, Jackson ImmunoResearch Europe Ltd, Ely, Cambridgeshire, UK). The ECL images were recorded using an imaging system (Fujifilm™ LAS-4000; GE Healthcare, Life Sciences, Marlborough, MA, USA).

### 4.5. Mechanism of APS8 Cell Toxicity

We defined the dose–response curves of APS8 toxicity using the ApoTox-Glo™ Triplex Assay (Promega, Madison, WI, USA), which provides information on cell viability, cytotoxicity, and caspase 3/7 activation in single assay wells [42]. Following the manufacturer protocol, the A549 and HT29 cells were exposed to two-fold dilutions of APS8 from 10 µM to 20 nM final concentrations, for 6 h at 37 °C prior to the ApoTox-Glo™ assay. The cell fluorescence was first measured using a multi-mode reader (Synergy™ Mx; BioTek Instruments, Inc.) at the excitation and emission wavelengths of 400 nm and 505 nm, respectively, for viability, and at 485 nm and 520 nm, respectively, for cytotoxicity. Apoptosis was then quantified on the multi-mode reader (Synergy™ Mx) by addition of luminogenic caspase-3/7 substrate 8 h after APS8 treatment. We averaged the measurements from 2–3 wells for each illumination band and each APS8 concentration in two independent experiments. The values were corrected for background and control normalized to finally present the data as means  ± SEM (n = 4–6). We determined the concentrations of APS8 that provoked a 50% response (EC_50_) using nonlinear regression analyses with the GraphPad Prism software (La Jolla, CA, USA). These data were analyzed using bell-shaped curve parameters for apoptosis of A549 cells and cytotoxicity of HT29 cells, response curves with variable slope parameters for viability of both cell lines and for apoptosis of HT29, and as the sums of two Gaussian curves for cytotoxicity of A549 cells.

### 4.6. Microplate-Based Ca^2+^ Measurements

We determined the Ca^2+^-uptake for the A549 cells after the siRNA treatments (i.e., targeting CHRNA7; nontargeting negative control), using Fluo-4 Direct™ Calcium Assay kits (Molecular Probes™, Life Technologies, Eugene, OR, USA), according to the manufacturer instructions. The A549 cells were loaded with the fluorescent Ca^2+^ indicator dye Fluo-4 in the presence of 5 mM probenecid, for 30 min at 37 °C, and then left at room temperature for an additional 30 min. These cells were treated with various concentrations of the nAChR agonist ACh (100, 40, 20, 10, 5, 2.5, 1, 0.5, 0.1, 0.02 µM) and with APS8 (10, 5, 2.5, 1, 0.75, 0.5, 0.25, 0.1, 0.05, 0.01 µM). Alternatively, the A549 cells were pre-incubated with the indicated concentrations of APS8 for 20 min at room temperature, and then stimulated with 100 µM ACh. The fluorescence intensity increased in response to Ca^2+^-uptake was measured using a multi-mode reader (Synergy™ Mx;) at the excitation and emission wavelengths of 494 nm and 516 nm, respectively. Whole-well-based measurements were performed for the changes in intracellular Ca^2+^, in triplicate for each ACh and APS8 concentration. We corrected the mean values for background (i.e., for the fluorescence intensity in the wells without cells) and express the data as changes in relative fluorescence units (∆RFU), as the maximum response (F_max_) minus the minimum response (F_min_), divided by the minimum response (F_min_) according to the equation ($\Delta RFU=\frac{F_{max}-F_{min}}{F_{min}}$). Dose-dependent calcium response curves were obtained with 95% confidence intervals by nonlinear regression analyses of the ∆RFU, using the GraphPad Prism software (La Jolla, CA, USA).

### 4.7. Ca^2+^ Imaging with Confocal Microscopy 

A more detailed analysis was carried out for Ca^2+^ uptake in individual A549 cells using the Leica Confocal Software on an inverted multispectral laser-scanning confocal microscope (Leica Microsystems, Heidelberg, Germany) with argon laser excitation at 488 nm. In this analysis, we randomly selected 20 individual cells as regions of interest per experiment (this thus included between 80% and 100% of the total cell number in each field of view). Each experiment was repeated three times. Ca^2+^ imaging of the A549 cells was carried out using Fluo-4 Direct Calcium assay kits (Molecular Probes™, Thermo Fisher Scientific), according to the manufacturer recommendations. We used the following final concentrations of reagents: 600 nM APS8; 6 mM 4-azaspiro[bicyclo[2.2.2]octane-2,2’-[1,4]oxazolidine]-5’-one [43] (AR-R17779), a highly selective agonist of the α7 nAChRs; and 5 µM ionomycin, a specific calcium ionophore. The fluorescence emission was monitored at 500 nm to 540 nm. For each condition, the basal fluorescence (F_0_) was first measured for 20 s, then the relevant treatment was carried out. We monitored the changes in fluorescence for a period of 5 min (F). Alternatively, the fluorescence response was measured in A549 cells pre-incubated with 600 nM APS8 for 5 min at room temperature, and then stimulated with 6 mM AR-R17779. The Ca^2+^ uptake in response to various drug treatments was analyzed in R [44] and the data are presented using ggplot2 [45]. The time courses for each region of interest were shifted to set the time of application as t_0_, and the fluorescence intensities were normalized according to $RFU=\frac{F-F_{0}}{F_{0}}$, where F_0_ was determined from the fluorescence signal (10 s interval) prior to the treatment, and F was the fluorescence signal after the treatment. The normalized time courses were used to determine the maxima for each region of interest and under each condition. The differences between the independent groups (drug treatment) were tested using nonparametric Mann Whitney U tests (Wilcoxon Rank Sum Test), with the GraphPad Prism software.

### 4.8. Toxicity Study for Determination of LD_50_ in Mice

Twenty-one 8-week-old male BALB/c mice that weighted from 22 g to 28 g were obtained from the animal breeding house (Veterinary Faculty, University of Ljubljana, Slovenia) to determine the APS8 toxicity dose (LD_50_) and to select the window of potential therapeutic doses for further in vivo studies on the antitumor effects of APS8. APS8 was dissolved in 100 μL sterile 0.9% NaCl and injected into the right tail vein of the mice at the doses of 3, 5, 6, 7, 8, and 10 mg/kg (n = 3 for each), with saline-treated mice (*n* = 3) as the negative controls. Following the Russell and Burch 3R principles [46], the LD_50_ was determined according to the adopted OECD 425 Directive (OECD Guideline 425 ‘Acute oral toxicity; up and down procedure’). The mice were monitored for 24 h for signs of intoxication and lethality. Those that survived for 24 h were sacrificed using carbon dioxide, followed by post-mortem examinations. The LD_50_ of APS8 was estimated according to the method described by Reed and Muench [47].

### 4.9. Antitumor Effectiveness

Female SCID mice were obtained from Charles River SrL (Calco, Lecce, Italy). The mice were 8 weeks old at the beginning of the experiment, and weighed from 20 g to 22 g. Tumors were implanted subcutaneously in the right flank of the mice by inoculation of 100 µL saline solution containing 1 × 10^7^ A549 cells, or 5 × 10^5^ HT29 cells. When the tumors had grown 5 to 7 mm in diameter, the mice underwent the specific treatment protocols.

In the first treatment protocol, two different doses of 2 and 4 mg/kg APS8 were tested, injected either intratumorally (20 µL/tumor) or intravenously (100 µL/mouse) in the A549 tumor-bearing mice. In the second treatment protocol, the mice were treated with two cycles of therapy with 4 mg/kg APS8 injected intratumorally (Figure 5A). Briefly, on days 0, 6, and 12, APS8 was injected, and afterwards, when the tumors started to regrow and reached 30 mm^3^, the mice were retreated, following the same schedule as in the first cycle. We used the same protocol for the HT29 tumor-bearing mice, which served as controls, due to the very low levels of α7 nAChR in HT29 cells. We monitored antitumor effectiveness and systemic toxicity as described previously [48].

### 4.10. Histopathology

Tissue samples of the heart, lung, liver, spleen, and kidney, were collected for histopathology from the male BALB/c mice used in the toxicity study. In addition, histopathology was performed for the kidneys and for tumors from female SCID mice. Formalin-fixed or immunohistochemical (IHC) zinc-fixed (BD Biosciences, USA) paraffin-embedded 4-µm tissue sections (from BALB7c and SCID mice tissues, respectively) were stained with hematoxylin and eosin (HE) and examined under the light microscope (Leica DMLB or BX-51 Olympus, Wetzlar, Germany), to determine the toxic effects and possible side effects of APS8 in normal tissues and for tumor analyses. Samples for histopathology were coded with numbers and an experienced pathologist evaluated them blindly without previous knowledge of the treatment protocols. Lesions were graded according to the standardized grade scales [49]. In addition, HE tissue sections of the kidneys from tumor-bearing mice were examined by the same pathologist who evaluated the tissue sections in the toxicity study.

Histological analyses of the tumors excised from the SCID mice were carried out to determine the responses to APS8 at the cellular level. From each group, three to five mice were sacrificed at different times after APS8 treatment (Figure 4), and three consecutive 2-μm-thick sections were cut from the tumors. The first section was HE stained to evaluate the percentage of tumor necrosis. The other two sections were used for IHC staining. To determine apoptosis, we incubated the sections with rabbit monoclonal antibodies against cleaved caspase-3 (1:1000; Ca-3, 5A1E, Cell Signaling Technology, USA). We used A peroxidase-conjugated streptavidin–biotin system (rabbit-specific HRP/DAB detection IHC kits; ab64261, Abcam,Cambridge, UK) as the colorogenic reagent. To determine cell proliferation, we incubated the sections with a monoclonal mouse anti-human antibody against Ki-67 (1:200; clone MIB-1 M7240; DAKO, Glostrup, Denmark). The sections were stained on an automated slide stainer with an indirect, biotin-free system (Optiview DAB IHC Detection kits, Basel, Switzerland). 

Tumor necrosis was evaluated on whole tumor sections by three independent observers. A microscope (BX-51) connected to a camera (DP72 CCD; Olympus; Tokyo, Japan) was used to determine the numbers of Ca-3 and Ki-67-positive cells in five fields of view of a viable tumor tissue, for each slide.

### 4.11. Statistical Analysis

We tested all data for normality of distribution. Following one-way ANOVA, the statistical differences were calculated for the antitumor effectiveness of the different APS8 treatments at the tumor doubling times using t-tests. The SigmaStat statistical software (Sigma Plot for Windows, v. 12.5; Systat Software Inc., San Jose, CA, USA) was used for the statistical analyses, and p values <0.05 were considered as significant.

## 5. Conclusions

The present study clearly demonstrated that α7 nAChR antagonists, like APS8, trigger apoptosis and necrosis in exposed cancer cells and tumors by the inhibition of α7 nAChR. This inhibition prevents Ca^2+^ influx, subsequent antiapoptotic signaling and cell proliferation. The therapeutic efficacy of APS8 evaluated in SCID mice revealed promising antitumor activity in tumors expressing large numbers of α7 nAChR, without systemic and organ-specific toxicity. 

## Supplementary Materials

The following are available online at http://www.mdpi.com/1660-3397/16/10/367/s1. Figure S1. *qRT-PCR.tif*. Confirmation of *CHRNA7* target gene and *GAPDH* positive control gene silencing as determined by qRT-PCR. Bars represent fold change (FC), with FC_min_ and FC_max_ as errors based on two biological and two technical replicates. The data were processed using the Applied Biosystems DataAssist^TM^v3.01 software. Figure S2. *Kidney histology.tif*. Representative images of histopathological changes in the kidneys of mice acutely exposed to APS8. (A) Cross-section of nephrons in juxtamedullary part of the kidney cortex. (B) The same kidney area in control mice. (C, D) Cross-section and longitudinal section of deep cortical nephrons from APS8-treated mice (5 mg/kg). Arrows, swollen renal epithelial tubular cells. Scale bars, 30 μm. Figure S3. *Tumor curves iv vs it.tif*. Growth curves of individual A549 xenograft lung adenocarcinoma tumors. Tumor bearing mice were treated as the control (A) or with intravenous (B, C) or intratumoral (D, E) injections of APS8, at the two doses indicated. Figure S4. *Mice body weight.tif*. Body weight changes of SCID mice bearing A549 tumors after intratumoral (*i.t.*) and intravenous (*i.v.*) injections of APS8 at the indicated concentrations. Figure S5. *CHRNA7 staining.tif***.** Representative images for the expression of the α7 nAChR in HT29 (left) and A549 (right) tumors. Brown staining indicates positive reaction.
